# Supplementary figures and images for: The connecting cilium inner scaffold provides a structural foundation that protects against retinal degeneration
Source: PLoS Biol. 2022 Jun 16;20(6):e3001649. doi: 10.1371/journal.pbio.3001649 (PMC9202906; doi:10.1371/journal.pbio.3001649)

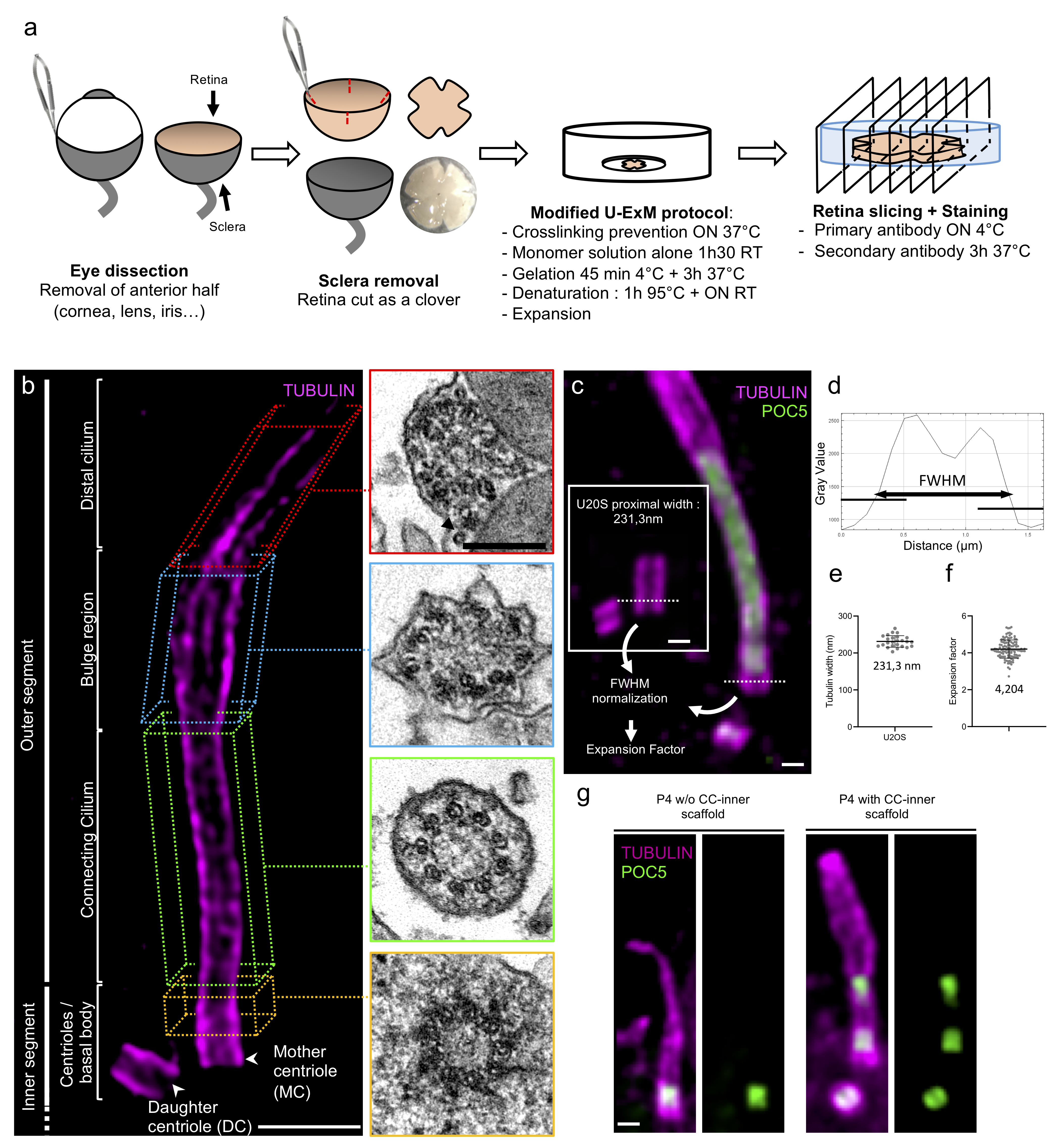

Supplement: S1 Fig — (a) Scheme summarizing the main steps of mouse retina dissection and expansion. (b) Expanded photoreceptor (left) highlighting the different regions of the distal inner segment and outer segment provided by the tubulin staining, together with the corresponding EM images (right). Scale bars: left = 500 nm; right = 200 nm. Black arrowhead points to microtubule singlet in the distal cilium. Note that the EM picture depicting the bulge region is the same as in the main figure. (c) Photoreceptor expansion factor calculation by comparing tubulin signal width at the photoreceptor mother centriole proximal end with the tubulin width of U2OS centriolar proximal end. Scale bars: 200 nm. (d) Fluorescence plot profile of tubulin at the level of the photoreceptor mother centriole proximal end generated with PickCentrioleDim plugin. (e) Measurements of U2OS centriole proximal width. N = 4 independent experiments. See S1 Table. (f) Distribution and mean of the expansion factors calculated from all the experiments performed on retinas. Note that developmental stage has no impact on the gel expansion; >40 experiments were used to measure expansion factor. See S1 Table. (g) Representative images of P4 photoreceptors with or without the CC inner scaffold. Scale bar: 200 nm. The data underlying all the graphs shown in the figure are included in the S1 Data file. CC, connecting cilium; EM, electron microscopy; FWHM, full width at half maximum. (TIFF) [file pbio.3001649.s004.tiff]

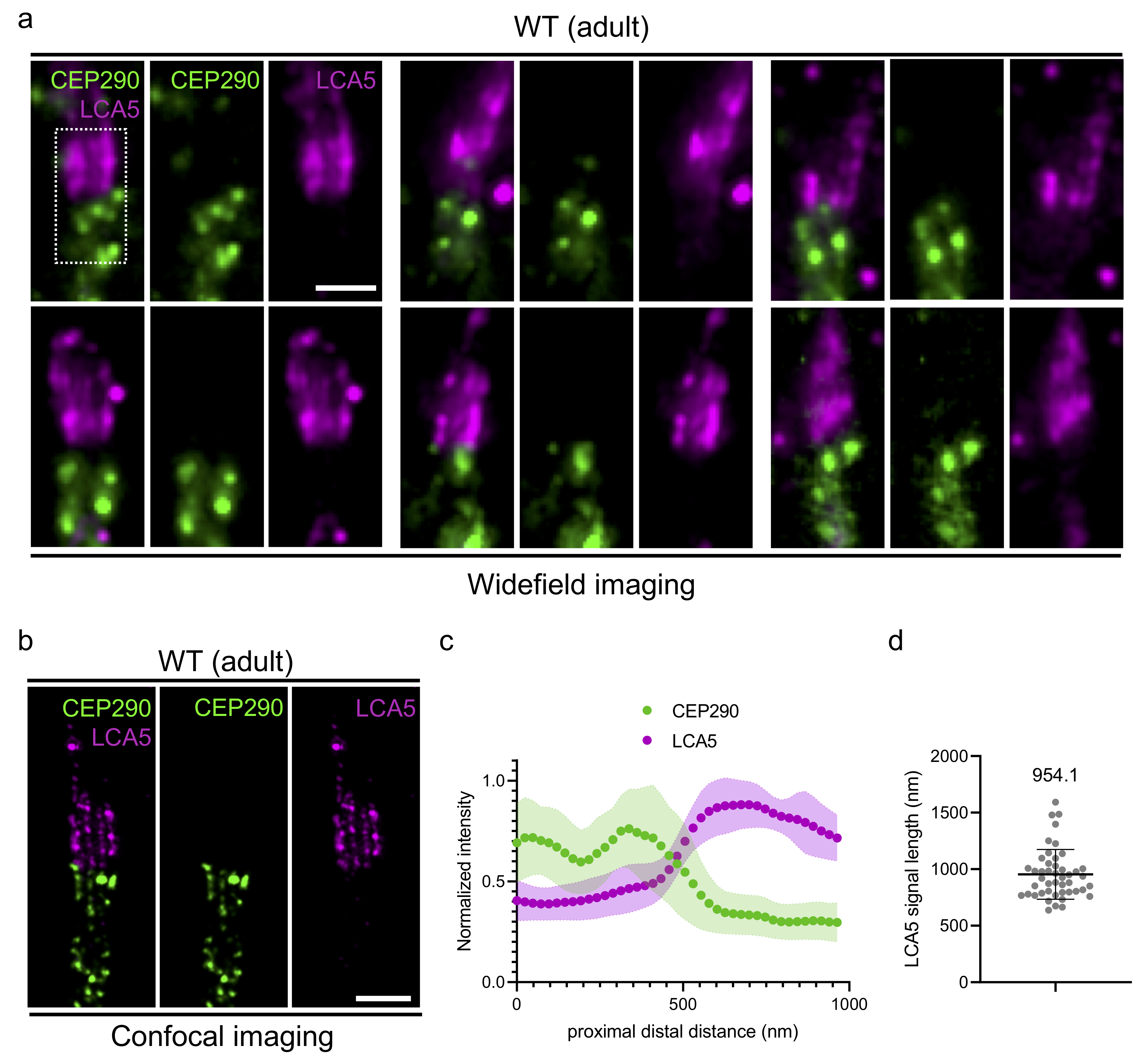

Supplement: S2 Fig — (a) Representative images of photoreceptor outer segments stained for CEP290 (green) and LCA5 (magenta). Dotted line rectangle represents an example of the region used in (c) for plot measurements. Scale bar: 500 nm. (b) Confocal image of a photoreceptor outer segment stained for CEP290 (green) and LCA5 (magenta). Scale bar: 500 nm. (c) Graph representing the average normalized intensity of CEP290 (green) or LCA5 (magenta) along the proximal to distal axis from 11 different pictures where examples are depicted in (a). Standard deviation is represented with transparent green (CEP290) or magenta (LCA5) areas. (d) Quantification of LCA5 signal length at the level of the bulge region in mature photoreceptors (≥P60). N = 3 animals. The data underlying all the graphs shown in the figure are included in the S1 Data file. MTD, microtubule doublet; WT, wild type. (TIFF) [file pbio.3001649.s005.tiff]

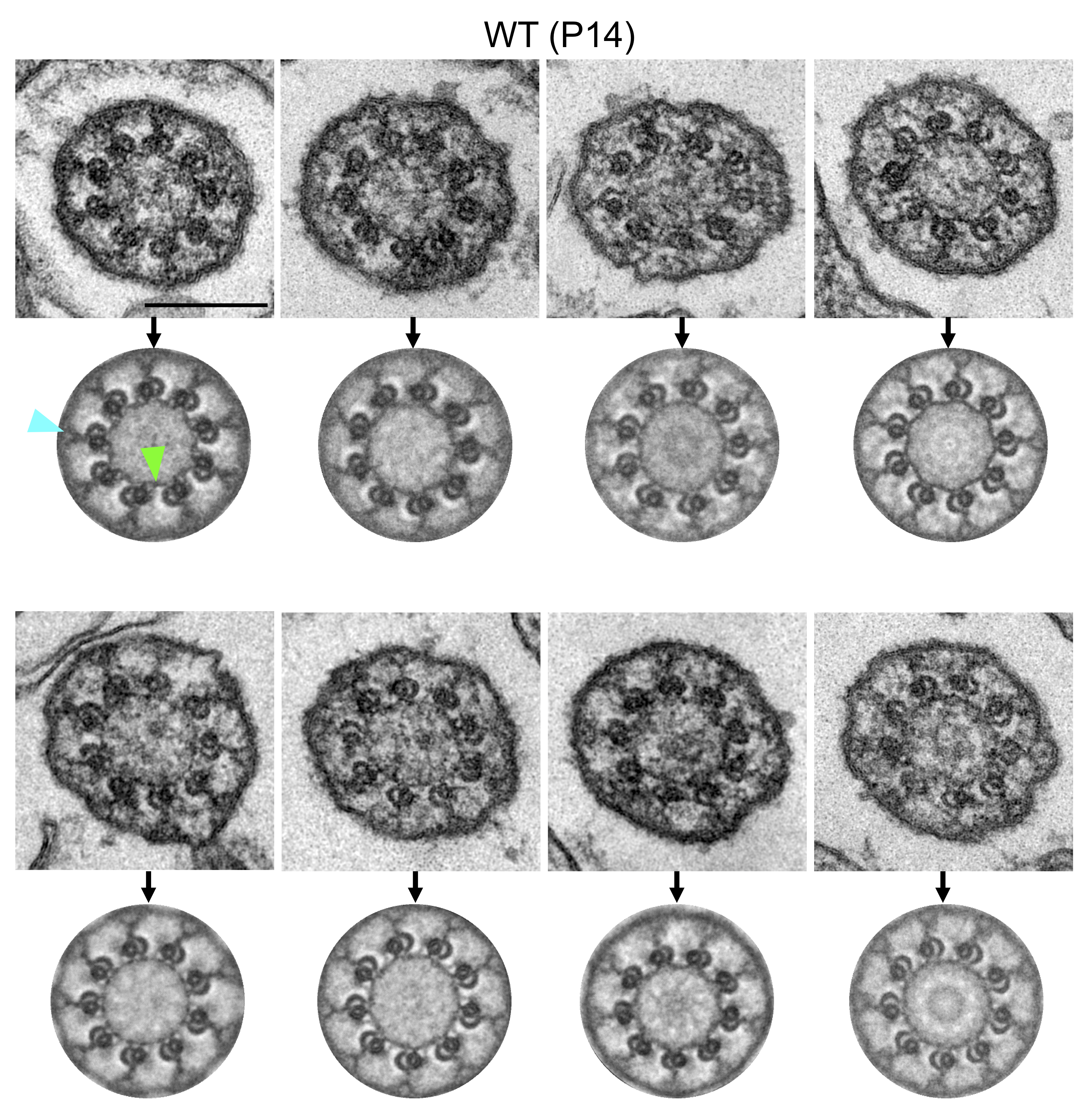

Supplement: S3 Fig — EM micrographs of WT P14 connecting cilia before and after symmetrization using CentrioleJ (see Methods), highlighting the presence of the CC inner scaffold (green arrowhead) and the Y-links (blue arrowhead). Scale bar: 200 nm. CC, connecting cilium; EM, electron microscopy; WT, wild type. (TIFF) [file pbio.3001649.s006.tiff]

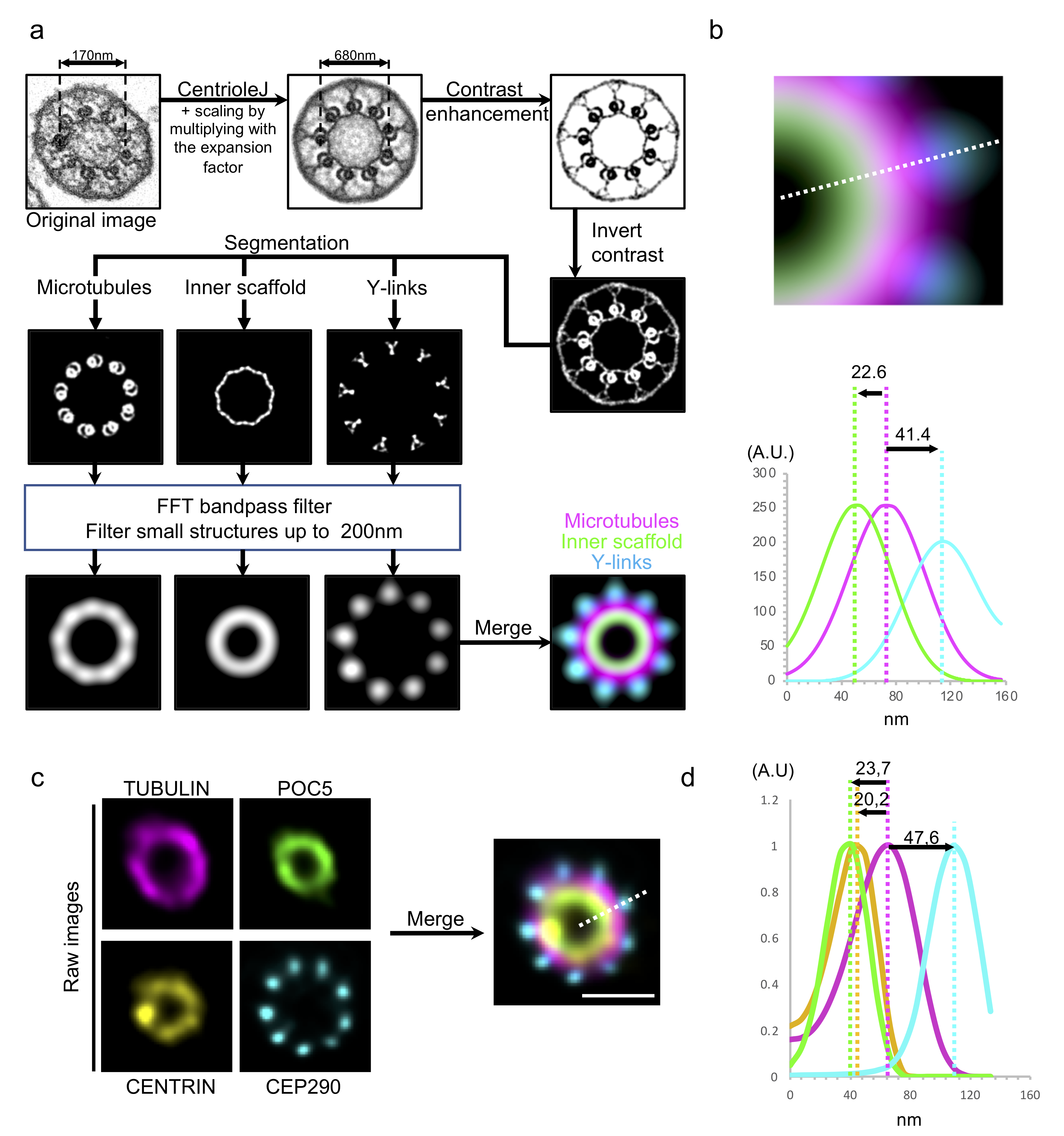

Supplement: S4 Fig — (a) Scheme representing the simulation pipeline as previously used in [16]. First, a raw EM micrograph was symmetrized using CentrioleJ (see Methods). Then, after contrast optimization, all the different structures (microtubules, inner scaffold, and Y-links) were segmented and submitted to a bandpass filter mimicking the limit of resolution obtained with classic fluorescence microscopy (200 nm). The simulated signals of each structure were then merged to reconstruct the final simulation. (b) Relative distance of each structure based on the reconstructed simulation. Top: representation of the line (white dashed) drawn to make the measurement of peak intensities for each structure (inner scaffold in green, microtubules in magenta, and Y-links in cyan). Bottom: peak intensity distances of each structure. (c) Merge of independent stainings of POC5 (green), CENTRIN (yellow), and CEP290 (cyan) to compare measurements with the simulation. The line drawn to make the measurement of peak intensities for each staining is represented with the white dashed line. Scale bar: 200 nm. (d) Peak intensity distances of each protein calculated from the merged image in (c). The data underlying all the graphs shown in the figure are included in the S1 Data file. EM, electron microscopy. (TIFF) [file pbio.3001649.s007.tiff]

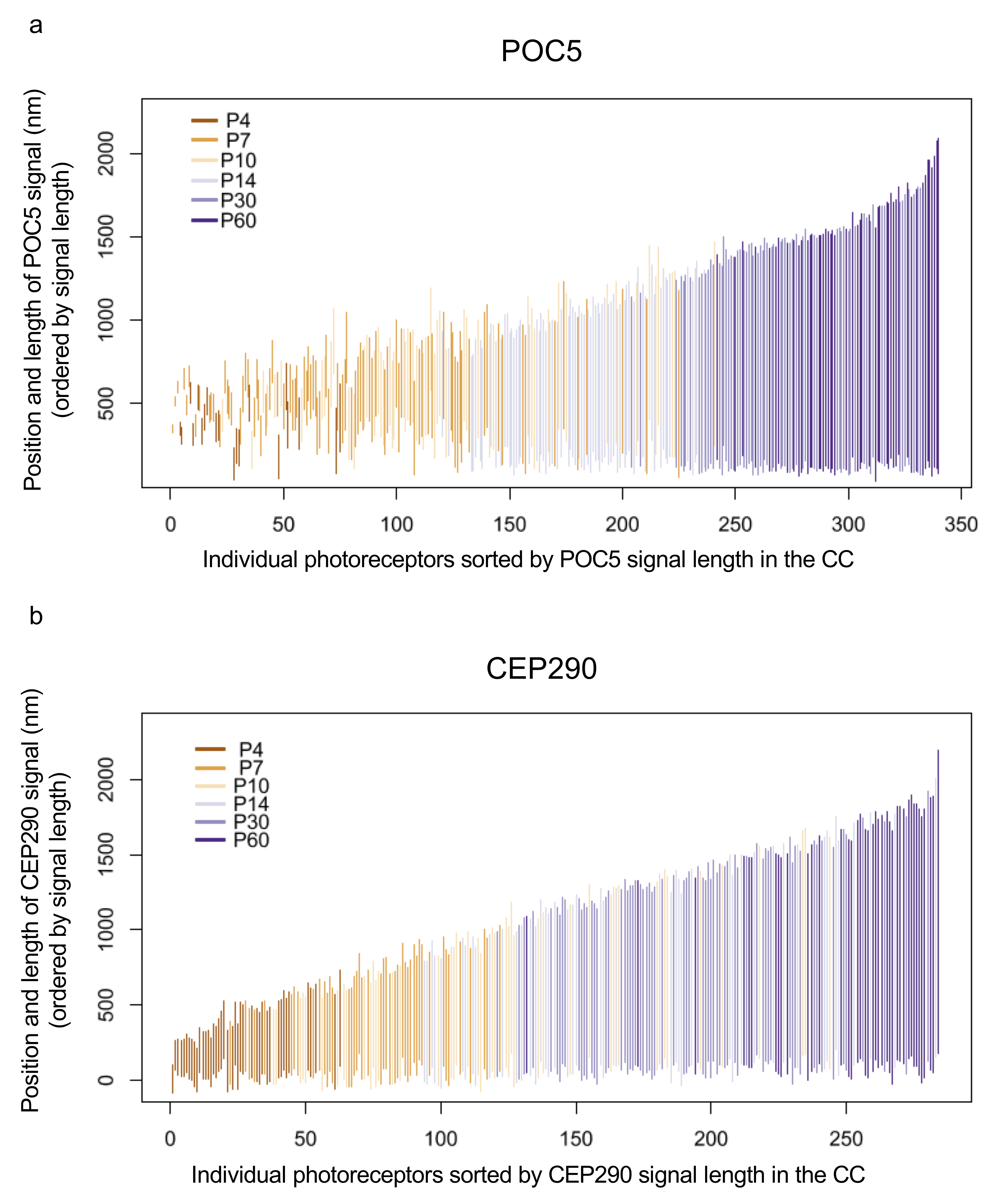

Supplement: S5 Fig — Graphs representing the position (compared to MC distal end as the “0”) and the length of the POC5 (a) and CEP290 (b) CC signals sorted by length. Each color depicts the age of the animals measured, confirming the timing of the growth of the 2 signals. The data underlying all the graphs shown in the figure are included in the S1 Data file. CC, connecting cilium. (TIFF) [file pbio.3001649.s008.tiff]

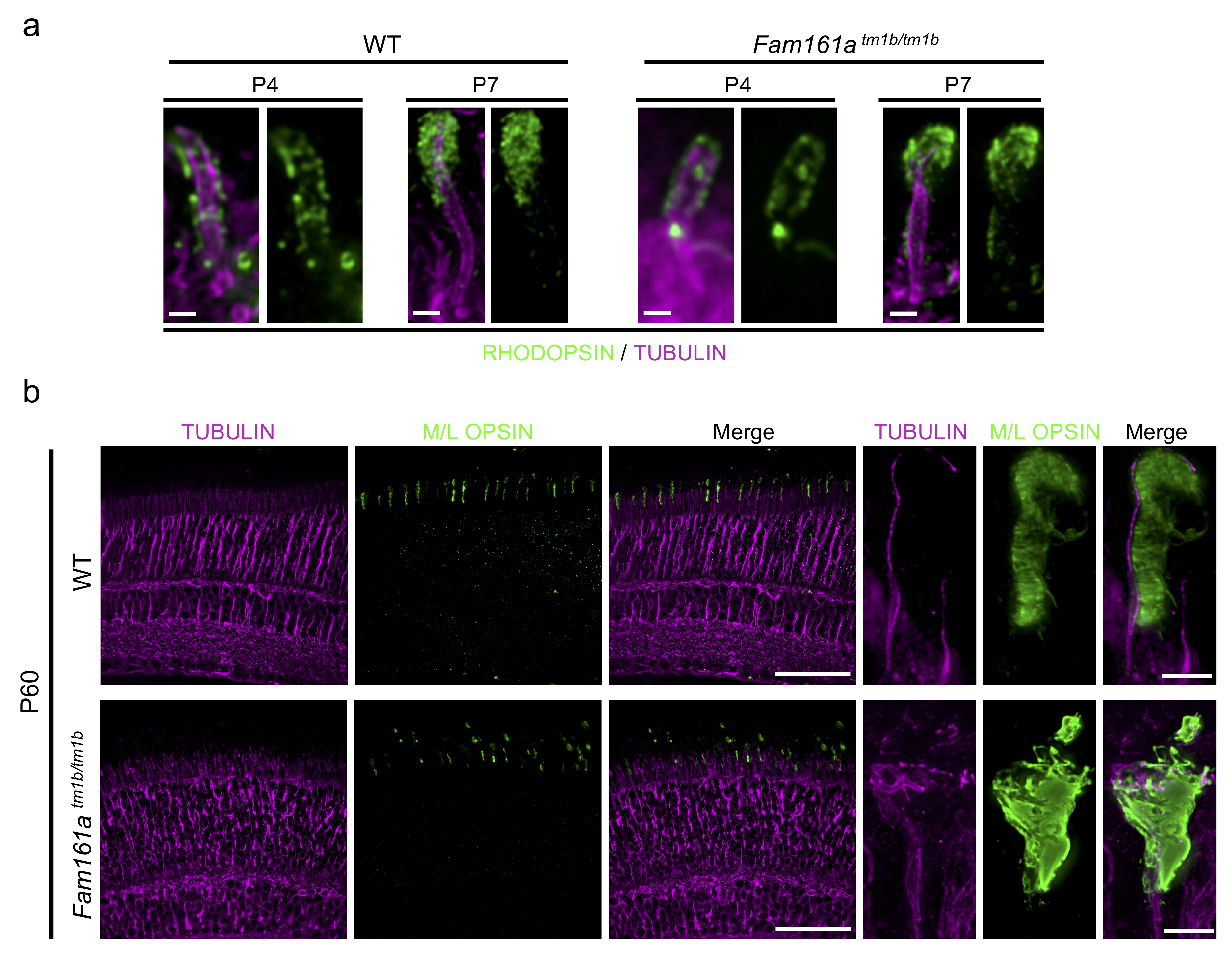

Supplement: S6 Fig — (a) Comparison of early rod outer segment development in WT and Fam161atm1b/tm1b photoreceptors. Note that RHODOPSIN outlines the outer segment tubulin signal at P4 and then accumulates distally at P7. Scale bar: 500 nm. (b) Expanded P60 WT or Fam161atm1b/tm1b retinas stained for CONE OPSIN (green) and tubulin (magenta). High magnification of the cone photoreceptors (right) shows that cone axonemes are also greatly impacted in Fam161atm1b/tm1b retinas, revealing no obvious difference between cones and rods at P60 in mutant mice. Scale bar: low mag = 50 μm; High mag = 2 μm. WT, wild type. (TIFF) [file pbio.3001649.s009.tiff]

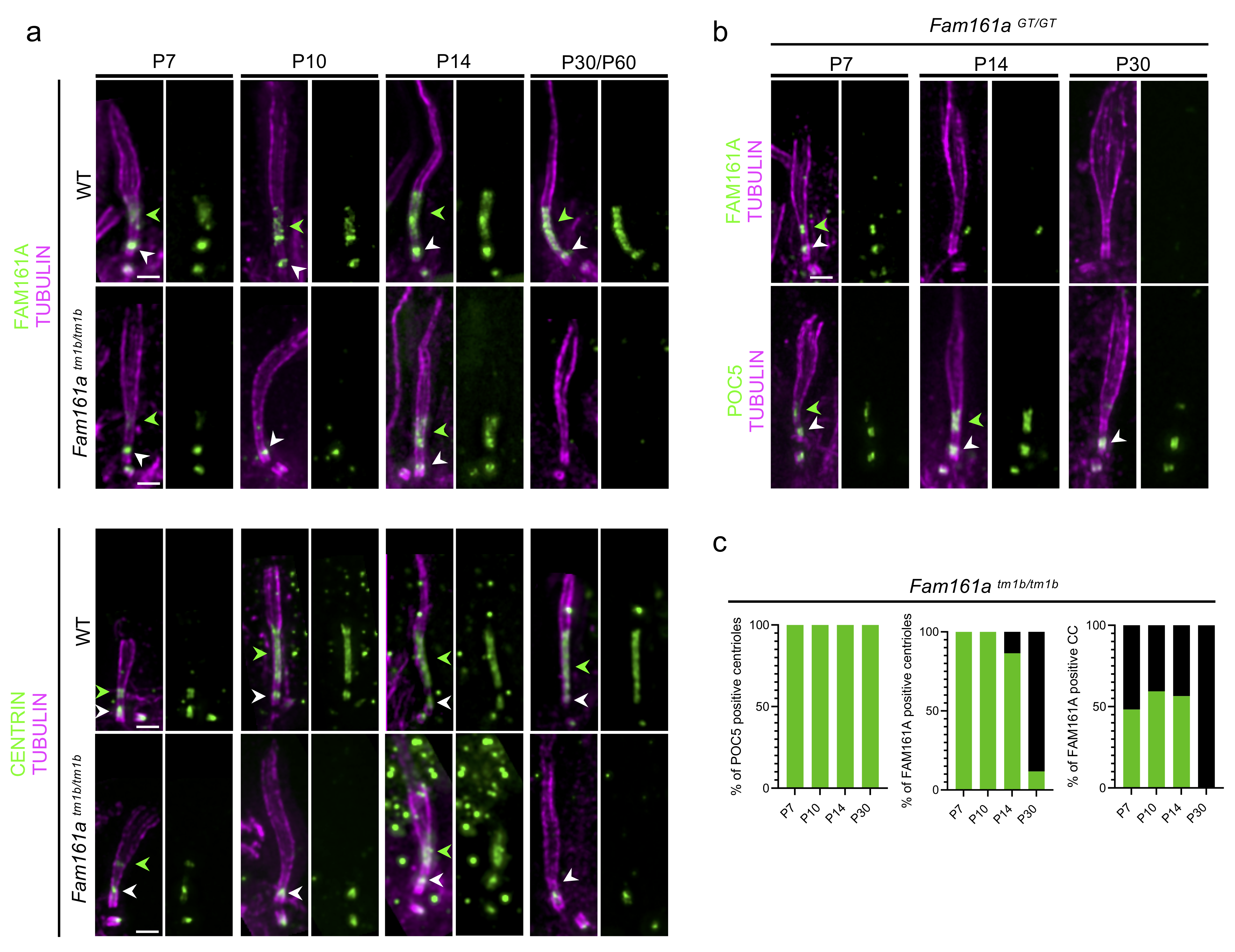

Supplement: S7 Fig — (a) Expanded WT and Fam161atm1b/tm1b photoreceptors stained for tubulin and FAM161A (top) or CENTRIN (bottom) at different ages. Note that P30/P60 represents a mixture of P30 or P60 images. Scale bar: 500 nm. White arrowheads point to centriole inner scaffold and green arrowheads point to CC inner scaffold, when present. (b) Expanded Fam161aGT/GT photoreceptors stained for tubulin and FAM161A (top) or POC5 (bottom) between P7 and P30. Scale bar: 500 nm. White arrowheads point to centriole inner scaffold and green arrowheads point to CC inner scaffold, when present. (c) Proportion of POC5-positive centrioles (left), FAM161A-positive centrioles (middle), or FAM161A-positive CC (right) in Fam161atm1b/tm1b photoreceptors between P7 and P30. Note, for comparison, that the data for the percentage of POC5-positive CC are presented in Fig 4I. The data underlying all the graphs shown in the figure are included in the S1 Data file. CC, connecting cilium; WT, wild type. (TIFF) [file pbio.3001649.s010.tiff]

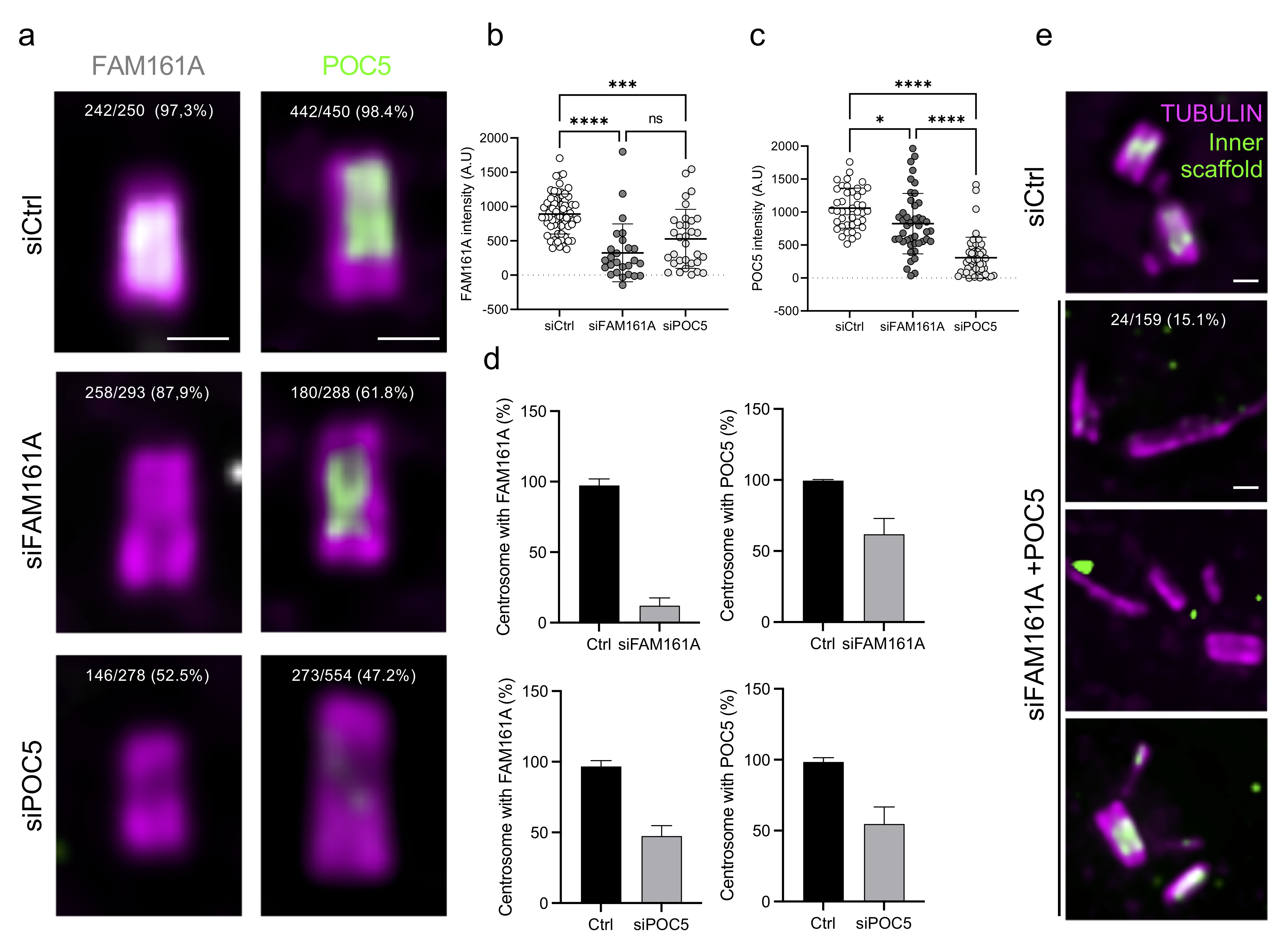

Supplement: S8 Fig — (a) Representative widefield images of expanded U2OS centrioles treated with siCtrl, siPOC5, or siFAM161A stained with tubulin (magenta) and FAM161A (gray) or POC5 (green). Inside each picture is depicted the total number of centrioles counted and the resulting averaged percentage from at least 3 independent experiments. Scale bar: 200 nm. (b) Mean fluorescence intensity of FAM161A under the indicated conditions. (c) Mean fluorescence intensity of POC5 under the indicated conditions. (d) Percentage of positive centrosomes (with FAM161A or POC5 staining) in siCtrl, siPOC5, or siFAM161A treated cells. (e) Representative widefield images of expanded U2OS centrioles treated with siCtrl or siFAM161A+ siPOC5 stained with tubulin (magenta) and inner scaffold protein (green). Scale bar: 200 nm; ≥3 independent experiments for each measurement. The data underlying all the graphs shown in the figure are included in the S1 Data file. siRNA, small interfering RNA. (TIFF) [file pbio.3001649.s011.tiff]

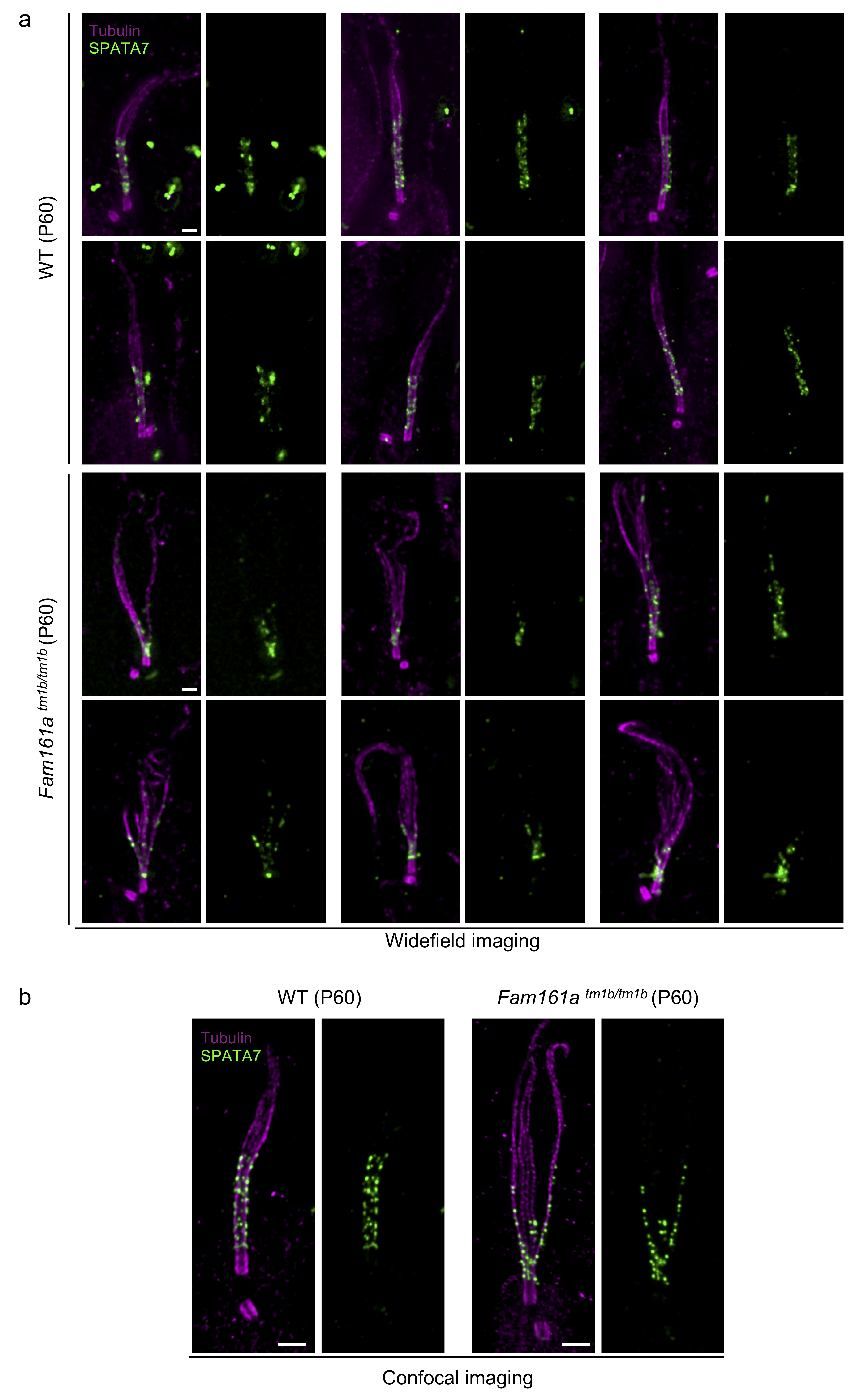

Supplement: S9 Fig — Representative images of widefield (a) or confocal (b) expanded photoreceptors from 2-month-old WT or Fam161atm1b/tm1b stained for SPATA7 (green) and tubulin (magenta). Scale bars: 500 nm. WT, wild type. (TIFF) [file pbio.3001649.s012.tiff]

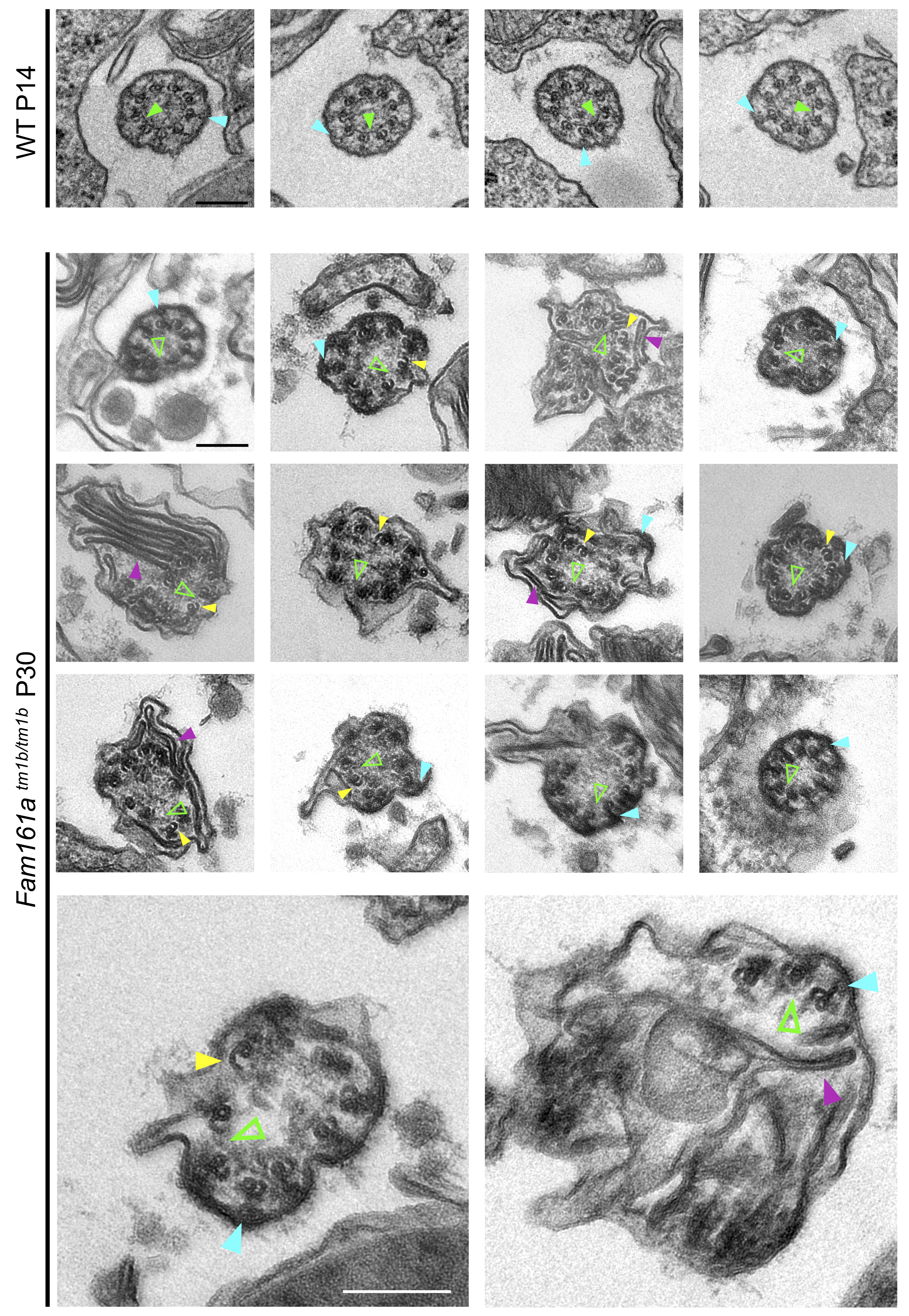

Supplement: S10 Fig — EM micrographs of WT and mutant connecting cilia revealing the loss of MTD cohesion within the mutant axoneme. Note that Y-links are still observable in some MTDs, even in strongly affected CC where membrane invaginations are present within the axoneme (bottom right). Filled green arrowhead highlights the presence of the CC inner scaffold. Empty green arrowheads indicate the lack of CC inner scaffold. Blue arrowhead highlights the presence of Y-links. Magenta arrowheads show the membrane invaginations. Yellow arrowheads reveal opened B-microtubules. Scale bars: 200 nm. CC, connecting cilium; EM, electron microscopy; MTD, microtubule doublet; WT, wild type. (TIFF) [file pbio.3001649.s013.tiff]

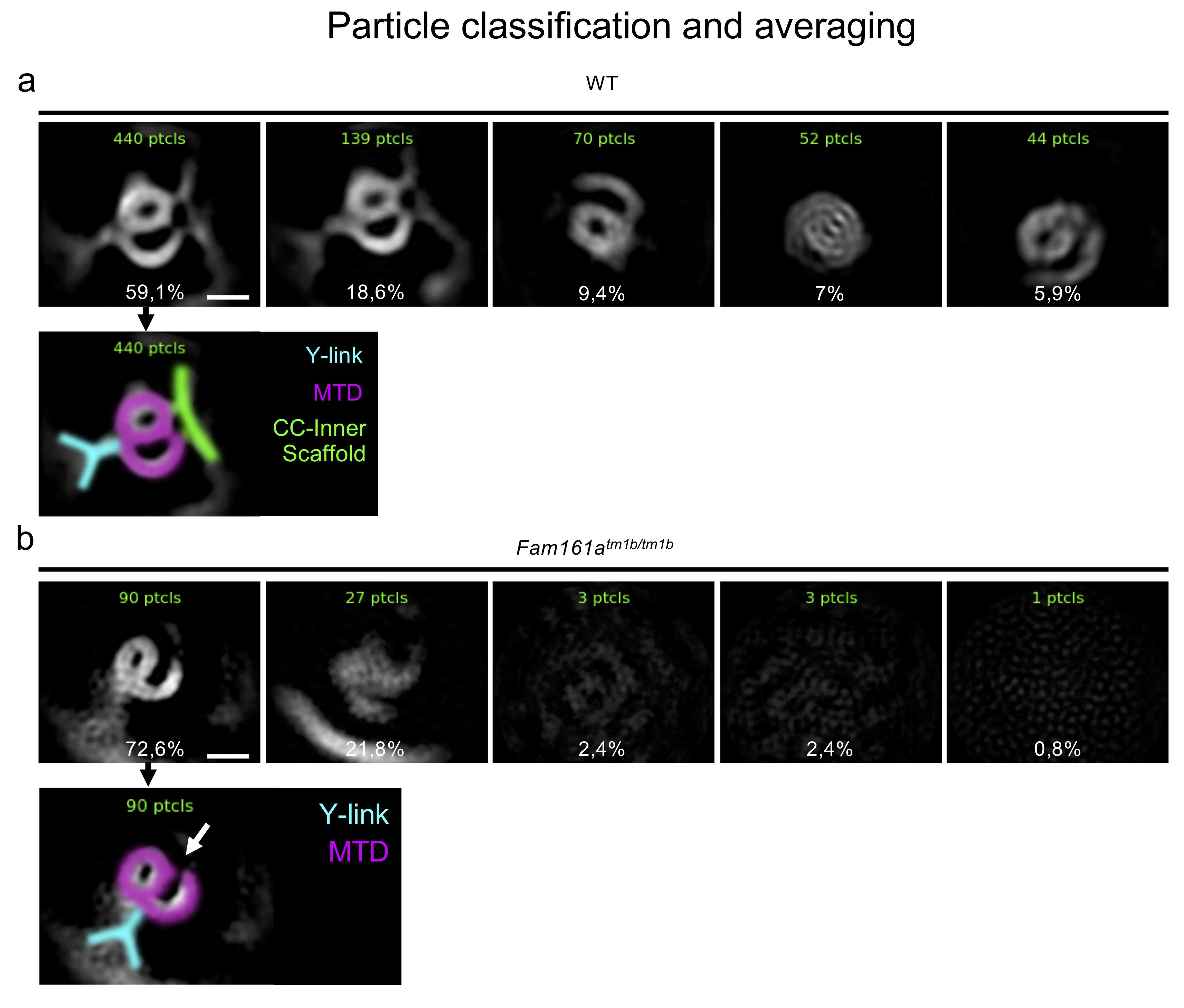

Supplement: S11 Fig — Representation of the classification (5 classes) of the particle averaging of microtubule doublets obtained from EM micrographs of P14 WT (a) or P30 Fam161atm1b/tm1b (b) (See Methods). The number of particles in each class is written in green, and the relative representation of each class is depicted below. For the most representative class of either WT or Fam161atm1b/tm1b, superimposition of the different structures (microtubules in magenta, Y-links in cyan, and the CC inner scaffold in green) was drawn for illustration purposes. Most representative classes were used for main figure. Scale bar: 20 nm. CC, connecting cilium; EM, electron microscopy; WT, wild type. (TIFF) [file pbio.3001649.s014.tiff]

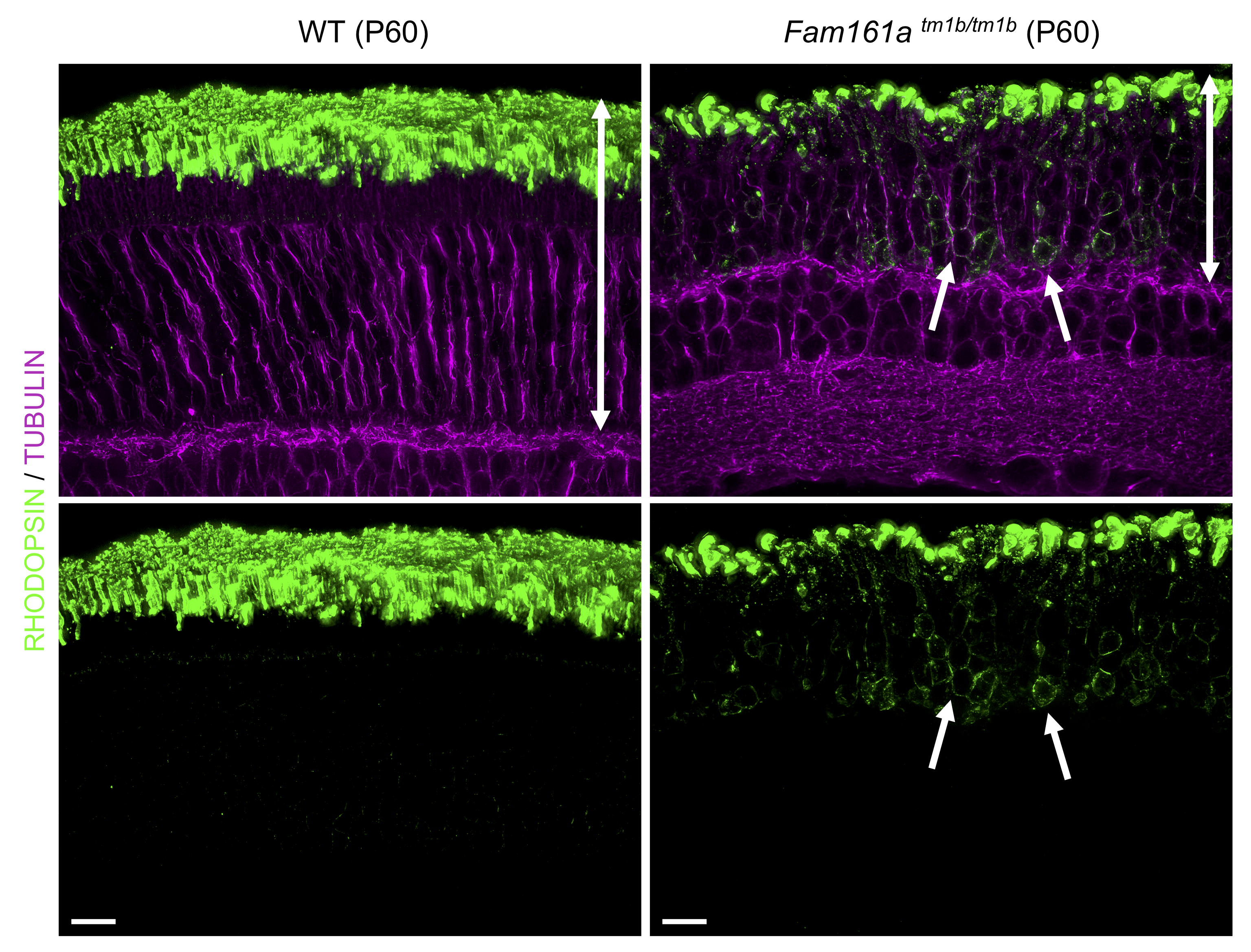

Supplement: S12 Fig — Expanded P60 WT or Fam161atm1b/tm1b retinas stained for RHODOPSIN (green) and tubulin (magenta). White arrows show RHODOPSIN signal at the level of photoreceptor cell bodies, notably around the nuclei in Fam161atm1b/tm1b retinas. Double-headed arrows reveal the difference of photoreceptor layer thickness between WT and Fam161atm1b/tm1b. Scale bar: 50 μm. WT, wild type. (TIFF) [file pbio.3001649.s015.tiff]
