# Supplementary material for: The connecting cilium inner scaffold provides a structural foundation that protects against retinal degeneration
Source: PLoS Biol. 2022 Jun 16;20(6):e3001649. doi: 10.1371/journal.pbio.3001649 (PMC9202906; doi:10.1371/journal.pbio.3001649)
Supplement: Attachment — Submitted filename: 121629_1_rebuttal_2279574_r6fb2r.pdf [file pbio.3001649.s016.pdf]

Full Revision

Manuscript number: RC- 2021-01126
Corresponding author(s): Virginie Hamel

1. Point-by-point description of the revisions

First of all, we would like to warmly thank the reviewers for their feedbacks and appreciate their comments and suggestions. We have made all efforts to address the comments and detail our replies in the point-by-point response below. Note that for the sake of clarity, we have highlighted the changes in the text in blue.

Reviewer #1 (Evidence, reproducibility and clarity (Required)):

In this study, Mercey and colleagues investigate the architecture and the role of proteins located in the connective cilium of mouse photoreceptors. Optimizing methodology to expand the size of the mouse retinal tissue (expansion microscopy), they show that POC5, FAM161A and CENTRIN proteins associate internally to the nine microtubule doublets along the length of photoreceptor connective cilium (CC). They call this structure « connective cilium internal scafold » (CC-IS) after the recent characterization of an internal scafflod constituted of the same set of proteins in centrioles of mammalian cells (Le Guennec et al, 2020). The presence of the CC-IS is further confirmed using electron microscopy. They show that the CC-IS associates early after CC formation and extends in a bidirectional fashion. Functionally, they show that mutations in Fam161a lead to specific disruption of the CC-IS after P14. Dislocation of the CC-IS is associated with microtubule doublets spreading and outer segment collapse, leading to PR degeneration, demonstrating its crucial role in stabilization of photoreceptor connective cilium and OS maintenance.

The existence of an internal scaffold containing POC5, CENTRIN1 and FAM161A proteins within the CC is strongly supported by both Ultrastructure-Expansion Microscopy (U-ExM) and electron micoscopy data. The results convincingly demonstrate that FAM161A is crucial for association and maintenance of CENTRIN1 and POC5 at the CC-IS and demonstrate its requirement for stabilizing microtubules of the CC. This is a very nice paper that will be of major interest to those in the field.

We thank the reviewer for careful reading of the manuscript and his/her supporting comments and suggestions to further improve the quality of our manuscript.

Major comments:

The authors show that the CC-IS develops around P4-P7 in the CC at a random location along the length of the nascent CC and show that the CC-IS grows in both directions, contrary to CEP290 structure, which protrudes unidirectionally towards the distal end of the CC, in continuity from the mother centriole. However, in Fig. 2b,c,h-j, the CC-IS stained by POC5

seems to consistently localize at the distal end of the CC structure, rather than at a random location. This seems corroborated by Fig. 3b, which shows POC5 signal directly below the bulge region (i.e. the end of the CC, characterized by an enlargement of the microtubule structure), at all stages studied, including P4.

We thank the reviewer for this important comment. Indeed, the way we introduced the random appearance of the CC- inner scaffold was misleading. The CC-inner scaffold, or at least the proteins described to form this structure, form at variable distances from the mother centriole, but not randomly along the CC. In fact, our results suggest that the CC-inner scaffold growth, and maybe other structures such as the Y-links, dictate the length of the CC, since the boundary between the CC and the bulge marked by LCA5 extends over time in parallel to both POC5 and CEP290 distal elongation (Fig 2f. and 2g). This is highlighted in Fig 3d, where we show that the end of POC5 signal coincides with the beginning of the bulge region where the axoneme diameter enlarges. We now have taken into account the reviewer's comment and clarified this notion in the revised manuscript stating (p. 8): "By measuring the relative position of the proximal and distal POC5 signal ends in the CC compared to the centriolar distal end, we also noticed that POC5 initially appears at variable distances from the mother centriole, while it appears below the beginning of the bulge region. We further found that its coverage elongates towards both the mature centriole and the distal end of the CC, suggesting that the CC-inner scaffold structure growth is bidirectional".

In addition, since the data of CC-IS development is descriptive, the data can only suggest a mechanism, and not prove it. The authors should therefore avoid overstating their conclusions on this point.

We apologize for the overstatement and have now tone down this statement in both the abstract and the discussion (p. 2 and p. 15-16 of the revised manuscript).
p. 2: "thus **suggesting** a molecular mechanism for a subtype of *retinitis pigmentosa*."
p.15-16: "Importantly, we associated the absence of a CC protein with the structural loss of the CC-inner scaffold, **allowing us to propose** a molecular and structural mechanism explaining this subtype of retinitis pigmentosa. »

Minor points:

-Could the authors add information on how retinas were sliced (what instrument, how thick, etc) and make it clear whether the slides were stained prior to or after slicing.

We thank the reviewer for this comment and apologize for not being sufficiently clear in the first instance. We now have added this information in the method section (p. 2 of the revised supplementary material): "Then, the gel was manually sliced with a razorblade to obtain approximately 0.5 mm thick transversal sections of the retina that were then processed for immunostaining. »

-Since the expansion microscopy method was optimized by the authors for retina tissue, it would

be important to indicate the variation of expansion factor observed between the different expanded retinas and indicate whether the developmental stage had any impact.

Once again, we apologize for the lack of clarity. The variation of the expansion factor observed for all the retina samples is depicted in Supplementary Fig 1f. In order to improve the clarity of the manuscript, we now clearly explain this in the legend together with the fact that the developmental stage has no impact (p. 10 of the revised supplementary material): "(**f**) Distribution and mean of the expansion factors calculated from all the experiments performed on retinas. Note that developmental stage has no impact on the gel expansion ».

-For top view EM acquisition of CC, could the authors clarify how they assessed the location of the section along the proximal-distal axis?

We now have added a sentence in the methods paragraph related to Electron microscopy (p. 6 of the revised supplementary material): "Location of the different sections along the proximal distal axis was determined thanks to the shape of the plasma membrane, and the presence of different structures (Y-Links, Inner scaffold). CC has round-shaped membranes, inner scaffold and Y-links whereas the bulge area has no Y-links nor inner scaffold and has a random membrane shape (due to the absence of the Y-links and the nascent OS disk formation)."

-Statistical analyses are largely adequate, but I suggesting adding in Table 1 how many animals were used per condition and wether the same number of measurements were used per animal for final statistical analysis.

This is a good suggestion. We have added a column in the table 1 in addition to the mention of it in the figure legends. Furthermore, we have added a sentence in the methods concerning statistical analyses for the number of measurements used per animal (p. 9-10 of the revised supplementary material): "Every mean, percentage, standard deviation, test and the number of animals used for comparison are referenced in Table 1 as well as in the figure legends. When possible, a minimum of ten measurements has been performed per animal."

-In Supplementary Figure7, it would be interesting to assess the percentage of PO5 positive CC in Fam161Atm1b/tm1b mice at P7, P10, P14, P30 to compare with % of FAM161A positive CC (similarly to Supplementary figure7 d). This would help determine whether the POC5 positive CC are in the same proportions as the ones positive for FAM161A. Quantification of the POC5 positive CC in the RP28 mouse model should be easily feasible, as the data already exist.

We apologize for not having been sufficiently clear. The percentage of POC5-positive CC in Fam161a$^{tm1b/tm1b}$ mice at P7, P10, P14, P30 can be found in Figure 4i. We now indicated in the figure legend the reference to the main figure : "Note, for the comparison, that the data for the percentage of POC5-positive CC are presented in Figure 4i.**"**

-Since the tissue was subjected to expansion, it would be appreciated if the authors could clearly indicate in the main text (rather then in the method section, as currently stated) when the distance units are adjusted by the expansion factor.

We thank the reviewer for this comment. We now have added a sentence in the main text to clarify this point (p. 5 of the revised manuscript): "For the rest of the study, all the measured data were corrected for the expansion factor."

-In the photoreceptor biology field, IS refers to the inner segment of photoreceptor cells. The use of the same acronym for the inner scafflod may be confusing. I wonder if the authors could not find a better acronym? Or even better, I would suggest not to use an acronym at all and use inner scaffold throughout the text.

We thank the reviewer for this important comment. We have replaced IS by inner scaffold throughout the manuscript and figures.

-Since Centrin is a family of 3 paralogs, I would recommend clarifying which of the 3 centrin protein the antibody labels (supposedly CENTRIN-1). Similarly, there are two different cone opsin paralogs in mice, the authors should indicate that the antibody was directed against Red/Green opsin.

We agree that these are important informations that were not clearly stated. We have now included these in Table 2. We used the 20H5 antibody that recognizes all centrins and the antibody AB5405 that recognizes the M/L opsin.

Suggested text changes:

Introduction

Thank you, we have corrected these two points as follows:

-MTDs extend distally to form the axoneme that stalks the OS rather than the OS itself.

Correction p. 3 of the revised manuscript: "This structural linker, emanating from a mature centriole, is made of nine microtubule doublets (MTDs) that extend distally to form the axoneme stalk, the basis of the OS.. .»

-It should be clarified that the incidence of 1/4000 relates to Retinitis Pigmentosa, and not to the RP28 subtype.

p.3 of the revised manuscript: "a subtype of *retinitis pigmentosa*, the most prevalent human inherited retinal disease with an incidence of 1/4000 worldwide ».

Results :

Figure 2b,e : the authors should add in supplemental figure1 an illustrative image of a photoreceptor at P4 with and without POC5 staining in the CC.

We understand the reviewer's point. We originally thought that, as an illustrative image of a P4 without POC5 staining is present in Figure 2b and another image of P4 with POC5 staining is present in Figure 3a, it would have been sufficient. However, to follow the reviewer's suggestion, we now have included this in the Supplementary Figure 1g for the sake of clarity.

As a matter of accuracy, in the schematic of Fig2i, the green region, depicting POC5 location should appear slightly higher than the region depicting CEP290 at all stages before P30 (Fig2j). Also, in the legend CEP290 is indicated in magenta, while it is in blue in the figure.

We thank the reviewer for this comment. We agree that, according to the Figure 2j, POC5 signal seems to appear a bit distally compared to Cep290 signal. However, these measurements (Fig 2h-j) come from a pool of all developmental time points, and the shortest POC5 signal depicted in Fig 2j are actually a mix of P4, P7 and P10 stages (See Supplementary Fig 5). That is why we decided to use the data from Figure 2f, where we see no significant differences between POC5 and CEP290 distal end signal at each time point, to illustrate the schematic in Fig 2l.

We have corrected the legend stating that CEP290 is in Cyan.

Please add a legend to the x-axis in supp Fig. 4.

We have added a legend as requested (now in Supplementary Fig.5).

The authors should add a graph to show how the distribution of classified particles compares between WT and Fam161A mutant mice. Also, the age of the animals used in this figure should be mentioned in the legend.

Thanks for this feedback. However, since the two classifications (WT or mutant) have been done independently, meaning that each class has been generated separately for the 2 groups and therefore are not exactly the same, it precludes the comparison of the two datasets.

We have added the age of the animals in the legend as requested.

Conclusions :

« CEP290 mutants still possess the CC-IS, confirming the independence of the two structures. » to what structures do the authors refer to? In the Potter et al study, the Y-shape links structure is not disrupted. The authors cannot therefore draw conclusions on the relationship between the CC-IS and Y-shape links structures but solely to the proteins themselves. This should be clarified.
We agree with the reviewer and corrected the sentence accordingly (p.18 of the revised manuscript): "Consistently, Rachel and colleagues showed that *Cep290*$^{ko/ko}$ photoreceptors still possess the CC-inner scaffold, confirming the independence of these two structures".

Reviewer #1 (Significance (Required)):

In the three last decades, work on human genetics and retinitis pigmentosa (RP) mouse models has led to tremendous advances in our understanding of photoreceptor sensory cilia. Importantly, a significant part of genes involved in RP are coding for proteins of the cilium, reflecting the crucial role of the connective cilium for photoreceptor development and maintenance. Despite this progress, the exact molecular composition of the substructures of cilium-associated structures remains unknown. For many ciliopathies, the primary defects leading ultimately to loss of ciliary function and photoreceptor cell death remain poorly understood. In this study, Mercey et al identify a set of proteins POC5, FAM161A and CENTRIN1 that locates internally to the connective cilium microtubule doublets. Reminiscent of the centriolar inner scaffold recently discovered, this structure is disrupted in mouse mutants for the Fam161a gene. In this model, microtubule spreading is observed with a shortened CC, and is associated with photoreceptor outer segments collapse, leading to progressive death of photoreceptor cells.

in this study, the authors used innovative (U-ExM) high quality tissue preparation for fluorescent and electron microscopy images to identifiy a novel architecture in photoreceptor connecting cilia, which are often affected in retinal dystrophies. Therefore, this work offers new insights into photoreceptor cell biology with potential impact in understanding disease pathology.

Scientists working on sensory systems and more specifically vision scientists and ophthalmologists should be highly interested in this novel findings. Cytoskeleton biologists should also be interested as this provide a new examples of such scaffolds.

We thank the reviewer #1 for the positive comments regarding our manuscript.

Reviewer #2 (Evidence, reproducibility and clarity (Required)):

In this work, Mercey and colleagues investigated the role of inner scaffold, structure they characterized in their previous work (Le Guenecc et al. 2020) for the formation of the connecting cilium in the photoreceptor cells, employing ultrastructure expansion microscopy they developed (Gambarotto et al. 2019) and electron microscopy of stained thin sections.

First based on their expansion microscopy of connecting cilia, they measured the distance from the center of the centriole to major basal-body proteins, centrin, CEP290 and POC5, demonstrating centrin and POC5 are located at the inner scaffold. This is the same result as their work on centriole from other species (Le Guenecc et al. 2020). They further extended observation longitudinally along the microtubule doublets and between various stages of connecting cilia growth. Interestingly POC5 was initiated at ~500nm from the proximal end and extends toward both proximal and distal directions during the ciliary growth. This leads them to hypothesize that the inner scaffold "zips" the nine microtubules into one bundled structure. They proved this hypothesis by examining the diameter of the cilia at and around the distal end of the inner scaffold and imaging the cross section by EM from Fam161, a mutant with defect of the inner scaffold. The defect influences the structure of connecting cilia especially after P30.

Concern: consistency with a past work

In Fig.3, MTDs spread (beyond the end of CC-IS) in WT CC. However, in the past cryo-ET study using the rod cell, CC seems straight without spreading (Gilliam et al. 2012 Cell 151, 1029). How do the authors explain this difference?

We thank the reviewer for raising this interesting comment. As we pioneered the use of expansion microscopy to study retina and especially photoreceptor cells, your comment concerning previously published high-resolution images, such as the pioneer paper of Gilliam et al., with Cryo-ET, is important. After digging into this publication, we noticed that, in Figure 3J and Figure S3, where the authors are looking at "the CC just below the region of nascent OS disk formation", the microtubules start to enlarge, similarly to our observation using U-ExM. In particular in Figure S3, microtubules, depicted in green and blue, are clearly wider in the upper part compared to the lower part. While the authors did not comment on this, we believe that their data and our U-ExM observations are very similar and consistent.

In the same paper, defect of the Cnbg1 channel protein causes the similar spread of MTDs. Is there any relation between CC-IS and this channel?

Once again, we thank the reviewer for raising an interesting point. While Cngb1 defects can lead to MTDs spreading, it is indeed not clear whether this is linked to the CC-inner scaffold or whether the observed defects results from an impairment of the above OS membrane structure (Fig 7j and K of the above-mentioned paper). However, authors did not mention any impact on CC. In addition, to our knowledge, no interaction has been shown between CC-IS proteins and the channel CNGB1protein.

Minor points:

Confusing terminology

"Tubulin diameter" (p7, p8), "tubulin spread" (p9) are confusing. "Diameter of cilia" and "MTD spread" will be more suitable.
We agree with the reviewer's comment and adapted the sentences accordingly : we changed for "axoneme diameter" and "spread MTDs" p. 9 of the revised manuscript.

Indication in Fig.3c ("Microtubule spread starts" with the purple double arrow) is unclear. Does MTD spread sometimes starts at the very proximal end of the centriole?

We thank the reviewer for pointing out this as indeed the original indication might have been misleading. The main message of the figures 3c,d is that the end of the CC POC5 signal coincides with the beginning of the bulge region where the axoneme diameter enlarges. To show that, we took the proximal end of the basal body stained with tubulin as a reference to measure i) the distance from the basal body proximal end to the CC-inner scaffold end and ii) the distance from the basal body proximal end to the position where MTDs spread. We have corrected the schematic to make this point clearer.

Supplementary Table 1:

The column of Figure 3b has no content. Maybe mistake during the editing.

Supplementary Fig.9:
It seems average from EM (Supp. Fig.8). But not explicitly mentioned.

We have corrected the two above suggestions. Note that now the supplementary for the EM average is supplementary Fig. 11.

Supplementary Fig.8:
The message of the 12 small panels (above) is not clear, while I imagine they want to demonstrate irregular shape, loss of IS and existence of Y-shape structure. Indications (like in the bottom two large panels) as well as images from WT controls are needed.
We thank the reviewer for this remark. We now extended the indications to all the images, highlighting the membrane invaginations with a magenta arrowhead, together with opened B-microtubules with yellow arrowheads and we have included four new WT images to ease the comparison (supplementary Fig. 10).

Reviewer #2 (Significance (Required)):

Findings in this work is highly novel and investigated in a multifaceted manner, presenting their argument convincingly.

We thank the reviewer for this very positive feedback.

Reviewer #3 (Evidence, reproducibility and clarity (Required)):

Major comments:
1.As indicated, the imaging presented is a major advance from regular confocal microscopy, and even challenges cryo-electron tomography and subtomogram averaging for certain details (while the practical procedure requires much less specific technical expertise and dedicated expensive equipment).
Nevertheless, a lot of previous work has been performed by several groups, using electron microscopy as well as cryo-electron tomography and subtomogram averaging (especially by the lab of Ted Wensel in Houston), to evaluate similar aspects of photoreceptor sensory cilium function in health and disease. One important study that was missed relates to the phenotype of Spata7 mutant mice, showing very similar microtubule doublet spreading and protein localization issues as the authors observed in the Fam161a mutant mice (REF: Dharmat et al, J. Cell. Biol. 2018; PMID: 29899041). These authors (in the lab of Ted Wensel) used cryo-electron tomography and subtomogram averaging, and indicated a proximal and distal connecting cilium region, which the current study further specifies. These results need to be critically compared and discussed by the authors, if possible experimentally in the FAM161A model.

We thank the reviewer for this important comment and we agree that pioneer works in electron microscopy have been done previously and should be compared to our results.

In the early time points of the *Fam161a*$^{tm1b/tm1b}$ mutant mouse (before P14), we indeed recapitulate a microtubule doublet spreading in the distal part of the CC, similar to what has been observed in the Spata7 mutant. This spreading then extends proximally at later time points to finally reach the distal end of the centriole. In the Dharmat et al. paper, all the analyses have been performed at P15, which is, according to the authors: “a time point that precedes the onset of photoreceptor degeneration, displaying a largely normal retinal histology with intact CC and outer nuclear layers”. So one could imagine that the distal phenotype observed in the Spata7 mutant could then propagate proximally in the same way as in the FAM161A mutant later on.

Following the reviewer’s suggestion to compare our data to the SPATA7 work, we analyzed by expansion microscopy SPATA7 localization both in WT and in *Fam161a*$^{tm1b/tm1b}$ mutant. Despite the low quality of the antibody staining that appears dotty, we could clearly show that SPATA7 localizes along the connecting cilium as expected from previous work. Moreover, SPATA7 signal appears mostly outside the microtubule wall, similar to CEP290 (Supplementary Figure 9). We next analyzed SPATA7 distribution in the *Fam161a*$^{tm1b/tm1b}$ mouse mutant model at P60 and found that SPATA7 localization is disturbed similarly to CEP290 signal (compare Fig. 4c to Supplementary Fig. 9). Once MTDs spread, SPATA7 signal lines the microtubules bundles, suggesting that it might bind the nine MTDs of the connecting cilium. The length of the SPATA7 signal seems to decrease under this condition similarly to what we observed for CEP290. Our current data with the markers analyzed do not allow visualizing distinct proximal and distal compartments along the CC but this remains a possibility that might be analyzed in future experiments. These additional data are now provided in the supplementary Figure 9 and stated in the text (p.12-13 of the revised manuscript).

2.The "Bulge area" (Page 4) seems to start where CC ends, but where exactly does it end? Is this based on solely the tubulin staining, until the point that the microtubules get more packed again or also based on the localization of proteins specifically to the bulge area?

We thank the reviewer for this interesting question. In the original manuscript, the bulge area was ill defined. We now clearly state in the revised manuscript that we can only define the beginning of the bulge area that correlates with a spreading of the MTDs as well as the presence of the LCA5 protein (p. 5-6 of the revised manuscript). Where does it end is still an open question as we could not identify clear end boundaries. However, in order to estimate the bulge region size thanks to LCA5 staining, we undertook the task to quantify LCA5 signal length in mature photoreceptors in the revised figures, and found that the signal spans on approximately 1µm (Supplementary figure 2d), that we proposed to represent the bulge region.

For example, lebercilin is indicated to localize here, but in the above paper by Dharmat et al., the region that they there call the DCC (distal connecting cilium) is maintained by SPATA7. How does this area relate to the RP1 protein, that is described to be located more distally to the CC? If possible, assess this experimentally.

In order to address this question, we bought an antibody against RP1 (HPA042257, Sigma-Aldrich) and assess its localization by expansion microscopy. Unfortunately, this antibody did

not work in U-ExM and we could not compare it to LCA5, nor SPATA7 or the bulge area. It is very well possible that RP1 could be part of this region but this remains to be determined. We are now referring to this possibility p. 6 of the revised manuscript : “We thus proposed that LCA5 delineates the bulge region (**Supplementary Fig. 1b**), but whether other proteins, such as the microtubule associated protein RP1, that has been described to be located more distally to the CC (Liu, 2004), also decorates this region remains to be determined.”

3."POC5 decorates entire CC region" (Page 4). If this is the case, what is the (small) area between the mother centriole and the POC5 staining? Is this small region not part of the CC?

Indicated in paper: We also noted a gap between protein signals in the centriole and the CC, suggesting that these two regions are independent
Page 7: POC5 signal then elongates in parallel to OS maturation until it reaches its final length of around 1500 nm at P60, similar to the described length of a mature CC (Rachel et al, 2015)

=> Can it be concluded based on the data in the paper that the gap is not part of either the centrioles nor the CC?

We thank the reviewer for this interesting question. We indeed suggested that the construction of the two inner scaffold structures (in the centriole and CC) is independent, because of the presence of this gap. Interestingly, in the centriole, the inner scaffold covers 70% of the total centriolar length and ends before the distal end of the centriole (Le Guennec et al, 2020, Figure 4d-h). This distal region devoid of inner scaffold is about 60nm long, which matches perfectly the gap observed in photoreceptor cells between the centriole inner scaffold and the CC inner scaffold. We therefore believe that this gap simply corresponds to the distal end of the centriole. We now clearly state this in the revised manuscript (p.5 of the revised manuscript): “We also noted a gap between protein signals in the centriole and the CC that we hypothesize correspond to the distal end of centriole devoid of the centriolar inner scaffold structure (Le Guennec *et al*, 2020), suggesting that these two regions are independent ».

4.Page 7: "We found that at P4, while all centrioles are POC5-positive and CC microtubules are already elongating, only 42% of photoreceptors displayed a POC5 staining at the CC" ◊ how are the authors sure that these are specific connecting cilium microtubules, how can these be distinguished?

We thank the reviewer for this comment. Indeed, at this stage, they should be named axonemal microtubules. We have corrected the sentence: “We found that at P4, while all centrioles are POC5-positive and axonemal microtubules are already elongating, only 42% of photoreceptors displayed POC5 staining at the level of ciliary axoneme, that may represent the nascent CC” (p. 7 of the revised manuscript).

5.Page 11: Similarly to FAM161A, POC5 and CENTRIN proteins showed an early and transitory localization at the CC before disappearing later on in the majority of the photoreceptors observed, despite mutant animals being heterogeneously impacted over time (Fig.

4b, f, i and Supplementary Fig. 6).
=> Fig. 4i shows that at P30 more than 50% of the CC still have a POC5 staining, while no CC POC5 staining is shown in the picture (Fig. 4b). The same is true for P14 and P60.
The authors comment on this by writing mutant animals being heterogeneously impacted over time.
Does this mean that some animals are more impacted than other animals, so that in one mouse there is still CC POC5 staining, while in the other mouse not? Or does this mean that in the same mouse some photoreceptor CC are affected, while others are not. Please clarify this.

We thank the reviewer to help us clarifying this point. The heterogeneity comes from differences between animals, but the data obtained from a single animal are homogeneous. This is exemplified in Figure 4f, where one mutant animal at P60 is barely touched in term of POC5 signal length (top red circles), whereas the other animals are greatly impacted with almost no signal inside the CC (lower red circles). We have now specified this in the legend of Figure 4i "Note that the heterogeneity is found between animals and not within a single animal » p. 28 of the revised manuscript. Moreover, we added the following sentence in the main text : "However, mutant animals were heterogeneously impacted over time, exemplified by the distribution of POC5 signal length within the CC between P14 and P60, explaining differences observed between POC5 and FAM161A stainings for late timepoints (Fig 4i and Supplementary Fig. 7c; p.11 of the revised manuscript)."

6.Both CEP290 and LCA5 show a 9-fold symmetry. Is LCA5 localizing in the extension of CEP290?

So if you look at the edge of the CC and the bulge, are both proteins detected at exactly the same location? Can this be distinguished/measured?

We thank the reviewer for this interesting point. To address this comment, we performed a co-immunostaining of CEP290 and LCA5. To do so, we used a new CEP290 antibody raised in mouse (see methods section p. 3-4 of the revised supplementary material), which gave the same localization profile as the one used in the other figures. We found that indeed LCA5 localizes in the extension of CEP290 but does not seem to overlap with it. We analyzed further the distinct localization pattern using a plot profile of the two channels following a proximal to distal axis (Supplementary Figure 2a-c) and confirmed that the two proteins do not overlap (Supplementary Figure 2c). This new data fully supports our findings of Fig 2g, demonstrating that, at each measured time point, the end of CEP290 signal coincides with the start of the LCA5 signal. These additional data are now provided in Supplementary Figure 2 and mentioned in the revised text p.6 "We further analyzed the distribution of CEP290 relative to LCA5 and found that neither protein overlaps, with LCA5 lining up the MTDs in the extension of CEP290 on a ~1µm long region **(Supplementary Fig. 2a-d**)."

Minor/textual comments:

7. The abbreviation "IS", for inner scaffold, is confusing in the context of the photoreceptors, as the same abbreviation is commonly used for "inner segment", the photoreceptor cell body.

We thank the reviewer for this comment shared with reviewer 1. We have now replaced IS by inner scaffold in both text and figures.

8.The abstract (p. 2) needs more accurate phrasing, e.g.:

-Retinal degeneration: the subject of the current research is inherited retinal degeneration (IRD).

We have replaced retinal degeneration by “inherited retinal degeneration”.

-IRD can be congenital (e.g. Leber congenital amaurosis) or progressive (e.g. retinitis pigmentosa).

To simplify and take into account this comment, we removed “progressive” in the sentence “Inherited retinal degeneration is a leading cause of human blindness due to loss of photoreceptor cells”.

-The photoreceptor sensory cilium consists of the photosensitive outer segment, the connecting cilium and the ciliary basal body. The connecting cilium bridges the photoreceptor outer segment and the photoreceptor inner segment (or cell body). This inaccuracy is repeated at the introduction (p. 3)

We apologize for this inaccuracy that we corrected as such: “These highly specialized ciliated cells are partitioned into two main regions, a photosensitive outer segment (OS) and a photoreceptor inner segment, which are connected via a thin bridging structure known as the connecting cilium (CC) with its underlying ciliary basal body” p. 3 of the revised manuscript.

-a lot is already known of the composition, assembly and function of the connecting cilium, but much less is known about the dynamic mechanisms of proteins localizing to this subciliary region.
We have changed the abstract as follows: “While structural defects of the CC have been associated with retinal degeneration, its nanoscale molecular composition, assembly and function are barely known” P. 2 of the revised manuscript.

-Human gene names should be indicated in capitals, Italic
We have now corrected this page 3 and 16. In the abstract, we were referring to the mouse gene mutation.

-Last sentence: does Fam161a refer to the mouse gene (only a knockout is tested, so no mutations) or the human gene mutations (then the phenotype is not confirmed or it should be indicated that it concerns mice in this case.

Related to the above comment, we refer to the mouse deletion and not the human mutations. We thank the reviewer for spotting this and have corrected it accordingly "Furthermore, we show that *Fam161a* disruption in mouse leads to specific CC-inner scaffold loss and triggers microtubule doublet spreading".

-The proposed molecular mechanism concerns retinitis pigmentosa type 28 (RP28), as indicated in the introduction, as that is caused by FAM161 mutations: it is currently unclear if/which other RP subtypes are also associated to the indicated mechanism.

We have indicated that in the abstract: "suggesting a molecular mechanism for a subtype of retinitis pigmentosa".

9.Final paragraph of introduction is confusing (Page 3/4). First the authors describe the 4 proteins independent of the CC. After that, they hypothesize that these proteins are an important part of the CC.
Original: Besides being present at the CC, these four proteins are located at the level of centrioles, and compose the recently identified inner scaffold (IS) structure connecting neighboring microtubule blades => remove 'Besides being present at the CC'
Corrected: Recently, we identified these four proteins locating at the level of centrioles and composing the so called inner scaffold (IS), a structure connecting neighboring microtubule blades.

We really appreciate this suggestion and corrected it in the main text.

10.1st paragraph, 4th sentence of results (Page 4): To first validate it,.. => To validate our approach, ...

11.Supplementary 1b: thanks to the tubulin staining => provided by the tubulin staining

We have now corrected these two points

12.Elaborate (in discussion?) on the interaction between FAM161A and Lebercilin, mentioned in results. Based on the results of this paper => localization not overlapping. So, is the interaction still valid?

We are now discussing the interaction in the results and the discussion (p. 6 and 18 of the revised manuscript): "While we found LCA5 mostly enriched at the bulge region, we also found some weak localization that might explain the reported interaction between LCA5 and FAM161A. However, further investigations might be needed to elucidate this interaction at the level of the CC. »

13.Results describing LCA5 localization (Page 5): most of LCA5 lined MTDs in the bulge region, occasionally being weakly present at centrioles and along the CC. => LCA5 is also present at distal cilium, which is not indicated in the text.

This is now added in the text.

14.Page 6/7: We then investigated how and when the CC, and notably the CC-IS, assembles during early postnatal development of photoreceptors in mouse, between days 4 and 60 (P4 and P60)
=> P30 & P60 is not early postnatal anymore; Early postnatal is until ~P23. Ref: Brust, PMID: 26816516

We removed early and thank the reviewer for the correction.

15.Page 8: "To confirm this hypothesis, we measured between P4 and P60 the tubulin diameter at different positions along the CC photoreceptors" => should be: Photoreceptor CC

We have changed the text as follows: “To confirm this hypothesis, we measured between P4 and P60 the **axoneme** diameter at different positions » p. 9 of the revised manuscript.

16.Page 8/9: Moreover, this region has a larger perimeter and a less circular shape, correlating the lack of cohesion between MTDs (Fig. 3f, g). => Can also be partially explained by the absence of Y-links.

We have added this in the text (p. 9).

17.Page 9: Finally, we tested whether the tubulin spread observed in some retinal degenerative diseases such as RP, ...
=> The next part also starts with 'Finally', so the 'Finally' mentioned here is not correct.
=> Ref is missing => in which (RP) cases is the tubulin spread shown before?

18.Page 9: For this purpose, we next took advantage of an RP28 mouse model deficient for FAM161A (Fam161atm1b/tm1b) => adjust

19.Page 10: In addition, we noticed that FAM161A seemed more stable at centrioles in Fam161atm1b/tm1b mice, before vanishing with solely 12% of FAM161A positive centrioles at P30 (Supplementary Fig. 6a, c).
=> I think the authors mean that FAM161A is more stable at centrioles compared to the CC, however the sentence can also be interpreted as FAM161A mice compared to WT mice => Rephrase.

We thank the reviewer for all these feedbacks. We have corrected the text according to these suggestions.

20.Page 10: We further validated this pattern of residual FAM161A signal in a second mouse model deficient for FAM161A (Fam161aGT/GT), known to generate a truncated FAM161A protein (Karlstetter et al, 2014) (Supplementary Fig 6b).
=> Both mouse models show indeed residual FAM161A expression. However, now it seems the

authors suggest that the centriolar and CC pattern is similar in both mouse models. This is not the case. In the GT/GT model the centriolar staining of FAM161A is not more stable compared to the staining at the CC. Both are lost already at P7.
=> Elaborate a bit more on the GT/GT model.

We thank the reviewer for this comment. We discuss further the differences between the two mouse models (p. 11 of the revised manuscript).

21.Page 10: We next monitored the localization of POC5 and CENTRIN during the development of photoreceptors in Fam161atm1b/tm1b or Fam161aGT/GT retinas (Fig. 4b, and Supplementary Fig. 6a, b). => Also indicate Supplementary Fig. 6b.

This has been changed.

22.Supplementary figure 7a can be confusing. The indicated percentage could be interpreted as percentage of overlap of POC5 staining with Tubulin staining.
=> indicate counts (e.g. 10/16=62,5%)
This has been now changed according to reviewer's suggestion and in now on Supplementary Fig. 8a et e.

23.Page 12: Consistently, axonemal perimeter distribution was largely heterogeneous in mutant photoreceptors, together with a less round shape, demonstrating that the lack of the CC-IS impairs MTDs cohesion and OS organization
=> Impaired OS organization cannot be concluded from figure 4. => remove
=> Can be concluded after explaining figure 5.

We have now removed this part and conclude only this from figure 5 as suggested by the reviewer.

24.Page 13: ... is connected to the rest of the cell body via a thin region of 1.5 µm long and 200 nm large, called the connecting cilium (CC). => large = in width

Corrected now in p.14 of the revised manuscript.

25.Page 13: ...,it is not surprising that the CC has been identified over the years as a hotspot for mutations associated with photoreceptor degeneration
=> CC cannot be mutated. Genes encoding for CC proteins can be mutated.

Corrected now in p.14 of the revised manuscript.

26.Page 14: ...suggesting that depletion of FAM161A could leads to the loss of the stem,...
=> remove 's' from 'leads'

27.Page 16: The CC has always been considered as a long transition zone (TZ) specific of the photoreceptor cells, => of = to

We have now revised the manuscript according to rewiewer's suggestions.

Reviewer #3 (Significance (Required)):

Main advance compared to existing knowledge:
The loss of structural cohesion in some subtypes of retinal degeneration has previously been observed, but this is the first study to show that loss of inner scaffold integrity in photoreceptors is the underlying defect. Also, the function of this scaffold as a "structural zipper" in the CC is an important finding that may provide future context to investigations of retinal degeneration.

Another important advance is the detailed description of expansion microscopy for retinal imaging, which could fuel many other molecular studies of photoreceptor cell biology.
Lastly, although the authors don't mention this explicitly, their data suggest that this extended stalk, the CC, which provides the link between the photoreceptor inner and outer segments, is a hybrid of centriole and ciliary transition zone structure.

* Audience:
Primarily research of inherited retinal degeneration, as the photoreceptor cilia are studied, but impact also extends to cell biology and cellular signalling in general, related to the role of cilia as cellular signalling hubs.

* Expertise:
Functional genomics of ciliopathies.

We thank the reviewer for his/her positive feedbacks about the manuscript and its underlying findings.
