# Supplementary material for: The connecting cilium inner scaffold provides a structural foundation that protects against retinal degeneration
Source: PLoS Biol. 2022 Jun 16;20(6):e3001649. doi: 10.1371/journal.pbio.3001649 (PMC9202906; doi:10.1371/journal.pbio.3001649)
Supplement: Attachment — Submitted filename: Point by point answer - Mercey et al.pdf [file pbio.3001649.s018.pdf]

Reviewers' comments

Rev. 1: Michael Housset and Michel Cayouette
The authors have addressed the major points that were raised in the initial review and their answers/additional data improve the manuscript. This is a very nice study with beautiful and convincing data that will be of great interest to the field. However, one of the authors' responses is debatable:

On page 17 of the revised manuscript, the authors corrected the reference of the Cep290 mutant to support their point of a potential independence between the Y-link structure and the CC-inner scaffold: "Consistently, Rachel and colleagues showed that Cep290ko/ko photoreceptors still possess the CC-inner scaffold, confirming the independence of these two structures".

In their paper, Rachel and colleagues show that connecting cilia fail to dock at the inner segment membrane in photoreceptors of Cep290ko/ko mice at P14 (Figure 4), but they also show that a 9+0 microtubule ring still assembles. As Rachel et al. did not stain for proteins specific of the CC-Inner segment (which was not known at the time) and considering the suboptimal resolution of their en-face EM sections, this data cannot be used to conclude about the presence or absence of a CC-inner scaffold in Cep290 KO mice. Additionally, in contrast to the Rachel et al study, nascent CC were shown to form in Cep290 KO photoreceptors at P10 by Potter and colleagues (2021). In this study, centrin was shown to decorate the nascent CC of Cep290ko/ko photoreceptors (Potter et al, 2021, Figure 6h).

Thus, the authors can only speculate on the interdependence of the Y-link and CC-inner segment scaffold as their presence has not been carefully investigated in Cep290 KO photoreceptors and should modify the text to that effect.
We agree with the reviewer’s comment and apologize for the overstatement. In our EM pictures, the Y-link structure is still present in Fam161a $^{tm1b/tm1b}$ CC while the inner scaffold is absent. We have now corrected the sentence as follows:
Consistently, Potter and colleagues showed that Cep290ko/ko photoreceptors still possess the CC-inner scaffold protein CENTRIN, suggesting that CEP290 and the CC-inner scaffold are probably independent.

Minor comment on the revised manuscript:
Page 17 "While we found LCA5 mostly enriched at the bulge region, we also found some weak localization that might explain the reported interaction between LCA5 and FAM161A". Which localization are you referring to? The CC-inner scaffold? This is not clear.
We apologize for the lack of clarity and we modified the sentence as such: “While we found LCA5 mostly enriched at the bulge region, we also found some weak localization at the level of the connecting cilium and the centriole that might explain the reported interaction between LCA5 and FAM161A”.

Congratulations on this beautiful work.

We thank the reviewers for their very positive comments and help to improve the manuscript further.

Rev. 2:
The authors addressed all the points raised by this reviewer. The manuscript was satisfactory revised. This reviewer agrees with publication of this manuscript for PLOS Biology.
We thank the reviewer for his/her support.

Rev. 3:
The authors have adequately addressed all of my previous comments submitted to Review Commons, both textually and experimentally, and have done a commendable and accurate job revising the manuscript accordingly. One minor comment: The titles of Table 1 and Table 2 are mentioned in the revised Supplementary material, but the tables are included in the main manuscript text. If this is supplementary information, they should be included there.
We thank the reviewer for his/her support and have addressed the minor comment.
